# Auto-Segmentation via deep-learning approaches for the assessment of flap volume after reconstructive surgery or radiotherapy in head and neck cancer

**DOI:** 10.1038/s41598-025-08073-4

**Published:** 2025-07-01

**Authors:** Juliette Thariat, Zacharia Mesbah, Youssef Chahir, Arnaud Beddok, Alice Blache, Jean Bourhis, Abir Fatallah, Mathieu Hatt, Romain Modzelewski

**Affiliations:** 1 Department of Radiotherapy, Centre François-Baclesse, Caen, France; 2 Corpuscular Physics Laboratory, IN2P3, Ensicaen, CNRS UMR 6534, Caen, France; 3 University of Normandy, Caen, France; 4 LITIS - UR4108 - Quantif, University of Rouen, Rouen, France; 5 Nuclear Medicine Department, Henri Becquerel Center, Rouen, France; 6 Image Team GREYC-CNRS UMR, University of Caen, Caen, France; 7 Institut Curie, PSL Research University, University Paris Saclay, Inserm LITO, U1288, Orsay, France; 8 University Hospital, Amiens, France; 9 GORTEC. 4bis rue Emile Zola, Tours, France; 10 LaTIM, INSERM, UMR 1101, Univ Brest, Brest, France; 11 Laboratoire de physique Corpusculaire IN2P3/ENSICAEN/CNRS UMR 6534 – Normandie Université, Caen, France

**Keywords:** Head and neck neoplasms, Surgery, Flap, Volume, Neural networks (Computer), Oral anatomy, Cancer imaging, Oral cancer, Oncology

## Abstract

Reconstructive flap surgery aims to restore the substance and function losses associated with tumor resection. Automatic flap segmentation could allow quantification of flap volume and correlations with functional outcomes after surgery or post-operative RT (poRT). Flaps being ectopic tissues of various components (fat, skin, fascia, muscle, bone) of various volume, shape and texture, the anatomical modifications, inflammation and edema of the postoperative bed make the segmentation task challenging. We built a artificial intelligence-enabled automatic soft-tissue flap segmentation method from CT scans of Head and Neck Cancer (HNC) patients. Ground-truth flap segmentation masks were delineated by two experts on postoperative CT scans of 148 HNC patients undergoing poRT. All CTs and flaps (free or pedicled, soft tissue only or bone) were kept, including those with artefacts, to ensure generalizability. A deep-learning nnUNetv2 framework was built using Hounsfield Units (HU) windowing to mimic radiological assessment. A transformer-based 2D “Segment Anything Model” (MedSAM) was also built and fine-tuned to medical CTs. Models were compared with the Dice Similarity Coefficient (DSC) and Hausdorff Distance 95th percentile (HD95) metrics. Flaps were in the oral cavity (*N* = 102), oropharynx (*N* = 26) or larynx/hypopharynx (*N* = 20). There were free flaps (*N* = 137), pedicled flaps (*N* = 11), of soft tissue flap-only (*N* = 92), reconstructed bone (*N* = 42), or bone resected without reconstruction (*N* = 40). The nnUNet-windowing model outperformed the nnUNetv2 and MedSam models. It achieved mean DSCs of 0.69 and HD95 of 25.6 mm using 5-fold cross-validation. Segmentation performed better in the absence of artifacts, and rare situations such as pedicled flaps, laryngeal primaries and resected bone without bone reconstruction (*p* < 0.01). Automatic flap segmentation demonstrates clinical performances that allow to quantify spontaneous and radiation-induced volume shrinkage of flaps. Free flaps achieved excellent performances; rare situations will be addressed by fine-tuning the network.

## Introduction

Among patients diagnosed with head and neck cancer (HNC), more than 75% have advanced stages and are eligible for multidisciplinary management and combined treatments, including surgery and radiotherapy. Reconstructive surgery plays a crucial role in minimizing the functional and anatomical impact of tumor resection. It has become a standard approach across various cancer types to restore both structure and function. Even more critically than in breast cancer, owing to the vital structures of the head and neck region, HNC patients increasingly undergo reconstructive surgery using a flap to compensate for the tumor resection and associated tissue loss in the operative bed. Immediate HNC reconstructive surgery with a flap has become a standard in routine practice as it aims at alleviating the functional and aesthetic impairment associated with tumor resection. Flaps are now used in about 50% of operated patients overall with variations related to population treated and surgical expertise.

Flaps, which are composed of autologous tissues with an intrinsic blood supply, offer superior functional outcomes compared to grafts, particularly when addressing volumetric deficits. They are transferred from a non-cancerous donor site to the resection area, maintaining vascular continuity. Chimeric flaps combine bone and soft tissues to enhance reconstructive versatility, while bony flaps may be associated with hardware, such as metallic or carbon-fiber plates and screws, to restore bone integrity. The choice of flap is guided by the extent of the anticipated defect, assessed both clinically and through preoperative imaging (CT/MRI of the head and neck), along with patient-specific factors, including comorbidities and vascular integrity. Flaps are categorized into three main types: (a) local flaps, which involve repositioning adjacent tissue; (b) regional flaps, which rely on pedicled vascular rotation; and (c) free flaps, which involve microvascular transfer from distant sites. Flaps may be composed of a single tissue type, such as cutaneous flaps, or multiple tissue types, including fasciocutaneous, myocutaneous, and osteocutaneous flaps. The complexity of the defect dictates the choice of flap, and native tissues are not always replaced with identical tissues—for example, mucosa is often substituted with skin from the donor site. For about 10 years, head and neck flaps have been dominated by anterolateral thigh flaps, radial forearm free flaps, and pedicled pectoral flaps. Flap reconstruction is constantly evolving to enhance adaptability and functionality. Newer techniques prioritize versatile, composite, and customized flaps, and “free-style” flaps, which are tailored to highly specific reconstructive needs. Flap selection and shaping remains challenging as flaps inevitably undergo changes, such as flap shrinkage or fibrosis over time.

A recent meta-analysis showed that mean soft tissue shrinkage was of 37% and bone resorption of 14%^1^. However, flap changes are hardly quantifiable with accuracy at the individual level^2^; flap selection therefore remains empirical and lacks standardization. Flap segmentation could be used to monitor long-term flap changes from imaging. Artificial intelligence has indeed recently been proposed to monitor flaps postoperatively to detect early postoperative complications from patients’ photographs^3^, but flap segmentation, i.e. labelling/delineating flap borders on images, has not. When postoperative radiotherapy (poRT) is indicated, specific flap-related challenges are that flaps are most often not accounted for^4,5^ and heavily irradiated. poRT preparation relies on a postoperative CT but, while flap management during poRT preparation has recently been recognized as suboptimal by the radiation oncologists themselves^6^, manual delineation of a flap is a new tedious and challenging interdisciplinary task. Image labeling of the postoperative HNC anatomy is difficult due to postoperative inflammation, edema, lymphocele, wound healing scar, and surgical clips. Integration of a flap adds to the complexity of the radiological interpretation and flap delineation, as a flap corresponds to a missing anatomy situation, where ectopic reconstructed tissues of different appearance are placed. Labelling structures on images, i.e. delineation, has been a tedious and time-consuming task but has recently been substantially facilitated for standard organs using artificial-intelligence based tools integrated in routine planning softwares. The need for flap identification has been previously been recognized and several methods were proposed before the era of AI, such as clip marking of flap borders^7,8^.

The surgical literature suggests that flaps shrink and their functional outcomes deteriorate substantially more after radiotherapy^9,10^ than spontaneously with time. Longitudinal assessment is made difficult due to lack of easy flap volume measurement. To that aim, flap delineation is a prerequisite to quantify flap volume and texture. Flap delineation could allow to establish quantitative relations of patient characteristics, flap, and intervention with functional outcomes and causality of the various interventions.

In anticipation of the design of neural networks to automatically segment flaps in HNC, we have previously undertaken several steps. Manual delineation guidelines were established by head and neck surgical and radiation experts for the delineation of common soft-tissue flaps^11^. The validity and reproducibility of these guidelines were investigated using an interobserver study of more than 10 radiation oncologists. The Dice Similarity Coefficient (DSC), of 0.70^12^, was of the same order of magnitude as in international consensus for standard HNC tumor and organ delineation studies^13^. Automatic flap segmentation tools do not exist due to lack of databases of manually delineated flaps, the requirement of excessive use of clinician time, and the very challenging nature of the flap delineation task of missing anatomy/reconstructed tissue in the postoperative HNC setting. As now implemented for organ delineation in clinical routine^14^, automatic delineation of flaps could help to standardize contours, monitor flap changes and optimize poRT preparation processes.

Our aim was to develop an automatic flap segmentation model based on CT (as other images are not standard practice postoperatively) to be implemented in routine practice.

## Materials and methods

The imaging dataset was composed of postoperative radiotherapy planning DICOM RT data.

### Data

The automatic flap segmentation ancillary study was part of the GORTEC trial (NCT03576417). Out of 152 patients (33 health care centers) who had a flap visible on poRT planning CT between 2019 and 2021, 4 were excluded due to non-readability of DICOM data. poRT CTs are systematically calibrated for dose absorption (electronic density by Hounsfield units) and slice thickness is no larger than 3 mm. Other CT parameters were left to centre’s appreciation. There was no explicit mention of flaps and their management per standard practice at the time of study^4,5^. All flaps were accepted, dominated by anterolateral thigh flaps, radial forearm free flaps, and pedicled pectoral flaps, with a few outliers of various types according to the defect. Several situations are challenging for the automatic segmentation task and were retained in the database to be generalizable to routine use. At the image level, the muscle and fat have different densities and distinct Hounsfield unit values on CT. However, muscle and fat can be intermingled within the flap, as some flaps are shaped and folded onto themselves to conform to the recipient tissues, ensuring optimal integration. In such cases, the fatty component is generally surrounded by muscle, which helps provide structural support and maintain vascularization. Artefacts can be present due to metallic dental materials or plaques as they represent up to 75% of patients in daily care, with use of artefact minimization algorithms left to physician’s appreciation. Artefacts were graded *post hoc* by the experts involved in the delineation of flaps with respect to their impact on flap visibility (0-none, 1-light, 2-moderate, 3-severe). Flap location (oral cavity (OC), oropharynx (ORO), larynx or hypopharynx (L)), the type of flap (free flap or pedicled flap), soft tissue only or bone flap (+/- bone reconstruction), the presence of a feeding tube, bite block, lymphocele, lymphedema, tracheotomy, surgical clips, and contrast enhancement were assessed visually.

Flap contours were generated on treatment planning software Raysearch v8.0, according to flap delineation guidelines^11^ by two radiation oncologists (15 and 7 years of experience), involved in flap delineation training and an inter-expert reproducibility study^12^. Help from head and neck surgeons and a radiologist was obtained when needed. They were blind to patient characteristics, operative and pathological reports and outcomes. The final dataset was composed of 148 patients’ DICOM-RT CTs, structures (RS), including new flap contours, and dose matrices. Image characteristics available from tabular data and DICOM tags, such as voxel volume in mm^3^, were explored for their impact on model performances through cross-validation.

### Deep learning model for flap segmentation

We implemented a nnUNetv2 (‘no-new-UNet’) framework, the state-of-the-art approach for automatic segmentation of 3D medical images^15,16^ here to autosegment the full soft tissue flaps. The dataset was randomly divided into training and test sets of 120 and 28 patients’ CTs for full model validation but kept as 148 patients’ CTs for the cross-validation. Images were resampled to the median voxel size in the dataset (1.17 × 1.17 × 2 mm^3^). Images and Ground Truth masks were interpolated using 3rd -order spline and nearest-neighbor respectively. Data augmentation consisted of random flips, affine transforms (rotation, translation and/or scaling to the volume) of CTs and segmentation maps, brightness and contrast modifications, as well as CT image quality reduction. The nnUNet framework patch size (160 × 160 × 96) and batch size^2^ were used.

The method was further refined by developing a nnUNet framework to which Hounsfield Units (HU) windowing was added. In daily practice, experts use visualization windows, defined by a window level (WL) and a window width (WW) to distinguish between tumor and organs surrounding the HNC area. The reference method uses the 99.5 and 0.5 percentiles range of the HU values normalized by subtracting the mean from the image and dividing it by the standard deviation. This tends to squeeze different HU scales together, which is a problem in automatic flap segmentation, because the variation in HU between local soft tissues and flap tissue components can be small. To tackle this issue, we used a triple channel input instead of a single channel in the baseline. The first channel was the CT kept unchanged, the second one was clipped between − 180 and 260 HU before being normalized, the third one followed the same process but between 75 and 225 HU. This corresponded to WL = 40 and WW = 440, as well as WL = 150 and WW = 150 [18], creating better contrast for soft tissues, especially in the pharyngeal mucosa.

Finally, the recent 2D “Segment Anything Model” (MedSAM) framework based on transformers was used for comparison. It requires a user-defined input (i.e., region of interest containing the flap), before being fine-tuned onto medical images.

### Model performance in the testing dataset

We evaluated the global segmentation performance of each method on the aggregated validation sets using DSC, Intersection over Union (IoU) and Hausdorff Distance 95th percentile (HD95). The first two evaluate the overlap of the automatic and manual delineation while the latter evaluates the maximum distance between these. In addition, cross-validation ensured smoothing out any intra-dataset bias in the way the folds were split, since every case was used once for validation.

We also computed the relative and absolute differences in mean dose between the automatically and the manually delineated flap by reporting the flap delineated volume onto the planned dose map. This served to assess the clinical impact of segmentation accuracy.

### Impact of clinical and imaging characteristics on flap segmentation performances

In order to evaluate which clinical (such as free flap or pedicled flap, laryngeal or oral cavity primary, etc.) and imaging (such as the presence of dental fillings or reconstruction plaque creating severe artefacts blurring flap borders) characteristics could impair accurate model’s application in routine use, the DSC was obtained on each of the samples and studied in view of corresponding clinical and imaging characteristics of each case. Statistical correlations between these characteristics and the DSC were investigated using a Spearman rank test, requiring implementation of SciPy stats package for Python v3.11.

## Results

### Soft tissue flap characteristics

From the tabular data, the distribution of flap localization, composition and shape were analyzed (Table 1). Most flaps (*N* = 102) were situated in the oral cavity as in routine practice, 26 and 20 in the oropharynx and larynx/hypopharynx regions respectively. Most cases (*N* = 92) involved a soft tissue flap only (consistently with routine practice). In addition to their soft tissue component, 42 and 14 patients had a bone reconstruction, respectively with or without bone reconstruction. Most of the flaps were free flaps (*N* = 137, 92.6%), including 45 radial forearm flaps, 41 anterolateral thigh (ALT) flaps (of which there were 5 ALT-fibula flaps), 10 latissimus dorsi free flaps, 37 fibula flaps along with their soft tissue component, 2 jejunum flaps, 1 temporal free flap, and 1 infrahyoid flap. There were only 11 pedicled flaps (9 pectoralis major flaps, 2 latissimus dorsi pedicled flaps) in our dataset as in routine practice. This can be important later on as the shape of these flaps can differ from that of free flaps. The average flap volume was 60 cm^3^ based on ground-truth masks, ranging between 4 and 200 cm^3^.

**Table 1:** Patient, tumor, flap and image characteristics.

Characteristics	Number (%)
Flap site - Oral cavity - Oropharynx - Larynx or hypopharynx	102 (68.92) 26 (17.57) 20 (13.51)
Flap tissues - Soft tissue flap without bone resection - Soft tissue flap with bone resection without reconstruction - Soft tissue and bone flap	92 (62.16) 14 (9.46) 42 (28.38)
Flap type - Free flaps (45 radial forearm flaps, 41 anterolateral thigh flaps including 5 ALT-fibula flaps, 10 latissimus dorsi free flaps, 37 fibula flaps and their soft tissue component, 2 jejunum flaps, 1 temporal free flap, 1 infrahyoid flap) - Pedicled flaps (9 pectoralis major flaps, 2 latissimus dorsi pedicled flaps)	137 (92.57) 11 (7.43)
Mean, SD, (range), median	3, 8 (range:0–55), 1
Number of flaps per center - Low flap volume centers (1–2 flaps) - Intermediate flap volume centers (3–15) - High flap volume centers (> 15 flaps)	20 (60.60) 11 (33.33) 2 (0.07)
Proportion of flaps over operated patients: Mean, median	0.34, 0.27
Flap volume (cm3) (DICOM RT SS)	60.1, SD 42.7 (range:3.9- 199.8), median 50.7
High-dose PTV volume (cm^3^) (DICOM RT)	211.4, SD 171.5 (range:0- 1274.7), median 175.6
Low-dose PTV volume (cm^3^) (DICOM RT)	591.2 SD 260.4 (range:0- 1163.8), median 591.2
Percentage of flap volume in high-dose PTV (66 Gy) (DICOM fields)	68.02, SD 36.09 (range:0-100), median 82.86
Percentage of flap volume in low-dose PTV (54.45 Gy) (DICOM fields)	72.06, SD 39.23 (range:0-100) median 96.96
Tracheostomy (visual detection)	33 (22.30)
Feeding tube (visual detection)	26 (17.57)
Semi-quantitative aspect of metallic artefacts (visual detection) - Absent - Mild - Moderate - Severe	41 (27.70) 44 (29.73) 28 (18.92) 35 (23.65)
Surgical clips in operative bed (visual detection)	144 (97.30)
Lead marbles on mask (visual detection)	130 (87.84)
CT constructor - Scanner Type (DICOM tag 0008,1090) - GE o Discovery RT o LightSpeed RT16 o Optima CT580 o Optima CT660 - Philips o Brilliance Big Bore o Brilliance 16 - Siemens o Sensation open o SOMATOM confidence o SOMATOM go.Sim - Toshiba o Aquillion/LB - TomoTherapy (proprietary transformation) o Hi-Art o Accuray RingGantry	39 (26.35) 7 17 13 2 13 (8.78) 12 1 68 (45.951) 32 13 23 18 (12.16) 18 10 (6.76) 9 1
Contrast agent injection (visual detection) - None - IV (Arterial Phase) - IV (Portal Venous Phase)	106 (71.62) 34 (22.97) 8 (5.41)
CT acquisitions parameters in kV (DICOM tag 0018,0060)	139 (93.92%) at 120 (9 (6.08%) missing for Hi-Art CT)
XRay Tube Current (DICOM tag 0018,1151 )	196, 124 (range:0-594) median 165
Exposure in mA (DICOM tag 0018,1152)	132, SD 114 (range:0-668) median 96
Slice thickness z in mm (DICOM tag 0018,1050) (before resampling)	2.31, SD 0.45 (range:1.25-3), median 2
Spacing x or y in mm (DICOM tag 0028,0030) (before resampling)	1.277, SD 0.411 (range:0.586–2.734) median 1.172
Voxel volume in mm^3^ (x*y*z) (before resampling)	4.388 SD 4.084 (range:0.687–22.431) median 2.747

SD standard deviation, Gray Gy.

A feeding tube was visible in 18% of cases and a tracheotomy in 22%. Surgical clips embedded in the operative bed were always present excepted in 4 cases. 72% of the patients had some artifacts on their CTs, including 35 cases with severe artifacts, severity reflecting their impact on image blurring (both for the manual and automatic delineation tasks). Image voxel size was on average 4.4 mm^3^, with some low resolution CT scans exceeding 10 mm^3^. 28% of the planning CTs used for flap delineation and automatic segmentation were contrast-enhanced.

### Segmentation performance of the deep-learning segmentation algorithm

On cross-validation, the nnUNet achieved a mean DSC of 67.2% and a mean IoU of 56.4% for soft tissue flap segmentation. The nnUNet-windowing model performed slightly better with a mean DSC of 0.69 and a mean IoU of 58% (Table 2). Mean HD95 also improved from 28.1 to 26.6 mm with the nnUnet-window model, which showed robust localization of the flap, with a satisfying delineation performance, scoring similar as experts performance in their previous inter-observer study. The MedSAM framework obtained a mean DSC of 63%, and showed inconsistent segmentation results.

**Table 2:** Performance metrics for automated flap segmentation.

Metric	Dice Similarity Coefficient	Intersection Over Union (%)	Relative mean dose difference (automated vs. manually segmented flap) (Gy)
Mean	74.0	64.1	-0.68
Median	86.9	76.9	0.02
Range	0.0 / 93.4	0.0 / 87.7	-28.8 / 4.5

Gy Gray.

**Fig. 1:**
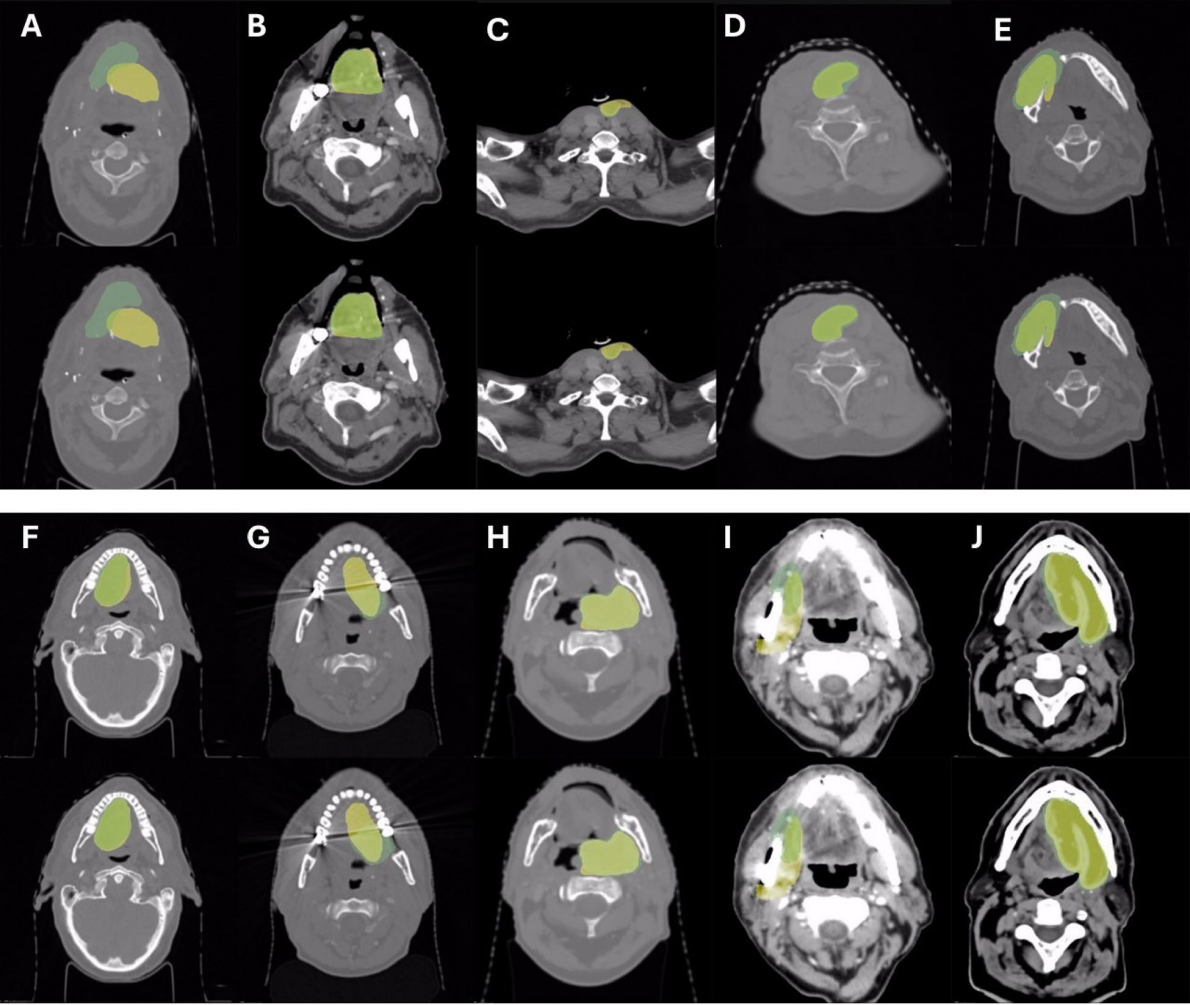
Visual examples of automatic segmentation by our model are provided compared to human contours.

The prediction and ground truth of the five flaps that achieved less than a DSC of 50% in at least two folds were further examined visually for interpretability (Fig. 1A-E). Suboptimal segmentation was either due to under-representation of some types of flaps (pedicled flaps) or due to image characteristics (artefacts).

Visual examples of automatic segmentation by our model are provided compared to human contours (Fig. 1). Example **A** shows an example of missed localization, in the oral cavity, probably caused by the very homogenous integration of the flap with the neighboring tissues. Example C shows a pedicled flap (underrepresented in the database and in clinical practice, as indicated for patients with significant comorbidities contraindicated for free flaps), where the flap’s body is well delineated, but the pedicled part is missed. Of note, manual delineation guidelines did not include the pedicle [10] and thus the ground truth did not either. Examples **E** and **I** demonstrate how the narrower window around soft tissues HU can help the delineation when a bone resection without reconstruction is present. Examples **B**, **D** and **F** display how our method improves the segmentation near the surface of the flap, which is important in a radiotherapy context. Example **G** is a relevant example of how artifacts can hamper delineation; in this case the upper part is missed due to image blurring.

### Statistical study of clinical and imaging characteristics

Characteristics were correlated to model performance (using DSC) including free or pedicled flap, associated bone resection/reconstruction, site of primary tumor (ORO, L, OC), image resolution as measured by the voxel size (VV) in cubic millimeters (mm^3^) and artifacts (A0-A3). Poorer model performances were driven by a highly modified anatomy due to bone resection without bone reconstruction (*p* = 0.0017); in contrast, flaps were correctly segmented in case of bone resection/reconstruction (no correlation observed with *p* = 0.7812). Severe artifacts were also strong drivers of inaccurate flap segmentation (*p* = 0.0049); the stronger the artifacts, the lower the DSC. Laryngeal flaps, in contrast to oral cavity/oropharyngeal flaps (*p* = 0.0094), and pedicled flaps, in contrast to free flaps (*p* = 0.0076), were driver of less accurate flap segmentation. The voxel volume was not statistically correlated with performance (*p* = 0.1323), showing that our model was robust to different image resolutions across the dataset.

As the CTs used in the segmentation framework were extracted from a trial database where poRT was performed, we examined how the flaps were managed by the radiation oncologists. A substantial proportion of the flaps were included in the high-dose radiotherapy region: median percentage of flap volume in the high-risk planning target volume of 66 Gy was 82.6% with many flaps having most this high-risk target volume visually centered onto the flap. Median percentage of flap volume in the low-risk target volume was 92.7%. Importantly, the relative mean dose difference between the automated and manually delineated flaps was of -0.68 Gy (range: -28.8 / 4.5). It was overall not clinically significant.

## Discussion

Although critical to improve functional outcomes and an area of considerable and continuous progress in reconstructive surgery, there are no accurate methods, to date, to quantify and predict flap volume and flap-related functional outcomes in the average and long terms. Similarly, flaps have been paid little attention at the poRT planning step until recently^6^. They are not delineated on postoperative images and significantly irradiated, agnostically, in most cases.

Our automatic flap segmentation method showed similar delineation performances for free flaps as those achieved among experts. Compared to a mean DSC of 0.70 for inter-observer variability of manual flap delineation by experts in the field in easier HNC tasks, such as the delineation of organs at risk or unresected tumors^12^, our study showed similar accuracy for the very challenging task of flap delineation in a highly modified and variable anatomy. We used a classical Unet framework, which remains the best to date for medical segmentation of 3D images, and added windowing that mimics the way radiological assessment is made by humans. We tested the MedSAM framework but the latter yielded significantly lower performances and was therefore abandoned. Transformer models are increasingly used in segmentation tasks with optimal outcomes, but do not seem to outperform the more classical nnUnet methods. However, the lower performance of the MedSam was possibly due to the 2D nature of the MedSAM method, unable to properly exploit the correlation between adjacent slices leading to large errors in parts of the flap volume from one slice to the next. Very recently, 3D MedSAM frameworks have been built but their performances remain to be shown. Also, nnUNet models may be less sensitive to limited dataset size such as in medical studies and in our case.

Importantly, contrary to many other computer vision studies, we did not remove artefacted images and outliers; as these represent typical clinical difficulties; so as to remain consistent and generalizable to real life. Finally, we used a cross validation to prevent biases in the way the folds were split, and therefore provide a realistic evaluation of the model performances, of 0.69. Delineation performances have been shown to achieve 0.71–0.74 for the 10 best algorithms in an international challenge but this was again for unresected HNC using multimodal imaging^17^, a much less challenging task than flap delineation. Recent advancements in deep learning have significantly improved the accuracy of segmentation in both skull bones and teeth, achieving Dice Similarity Coefficients (DSC) exceeding 0.9^18^. However, in the studies examined, CBCT images were used. Most importantly, CBCT images are were not appropriate for imaging soft tissues with sufficient resolution and for the segmentation of such complex soft tissues as flaps. Moreover, the articles examined were limited to relatively small datasets of about 50 patients, suggesting that the task of segmenting bone/teeth may be less challenging. One major challenge in segmenting teeth could be the presence of severe artefacts; however, the images shown suggest that artefacted images had not been included in the dataset. Our cohort was an unselected dataset of situations were metallic materials were present in over 50% of the patients and aimed to segment atypical soft tissues with standard CT. Moreover, every tissue (skin, muscle, fascia, fat, bone) or material could be included, per requirement of flap integration in the operative bed, making the task challenging, but more extrapolable to the variety of clinical practice.

The automatic segmentation method showed very good performances for free flaps. Limitations to this study include an imbalanced dataset, which however perfectly reflects practice. Pedicled and larynx flaps were underrepresented in the dataset (7% and 13% respectively). Pedicled flaps are usually reserved to patients with significant comorbidities and who cannot stand prolonged anesthesia. These rarer flaps were more prone to provoking errors in the automatic segmentation. Similarly, major anatomical modification, as represented by bone resection without reconstruction by a bony flap, was also underrepresented in the dataset (10% of flaps with bone resection left unreconstructed, 30% of bone flaps). All these characteristics are consistent with routine practice. Interestingly, the presence of a bone flap seemed to counteract the lower performance when bone resection was performed. Artifact severity negatively impacted automatic segmentation accuracy. Artifacts significantly blur images. Interestingly, most diagnostic and radiotherapy CTs now use algorithms that minimize artifacts (MAR). However, there were significant residual artifacts, that alter the information available for delineation by both the human expert and the model. We could not investigate the performances of MAR in the current work as use of MAR was not indicated in the DICOM fields^19^. More systematic and comprehensive tagging of DICOM fields of radiotherapy planning CTs should be encouraged not only for our specific task but also to improve quality and standardization of radiotherapy CTs. It should be noted that removing artefacted images from training datasets would limit the generalizability of the models. Current evidence for the use of artificial intelligence in reconstructive surgery is currently limited^20^ but should expand in the coming years in interdisciplinary areas of HNC care to improve patient outcomes overall such as is the aim of the flap segmentation project.

Finally, we have trained the first fully automatic segmentation method dedicated to flaps segmentation. This tool could help to predict spontaneous flap shrinkage over time and quantify flap shrinkage by fatty atrophy, conversion from muscle to fat, fibrosis, and correlations of such changes with treatment, including dose distribution patterns and functional toxicities. It might be integrated in flap selection algorithms in the future. Flap segmentation could also help to document and improve our understanding of the patterns of failure in the presence of flaps. In radiotherapy, this automatic segmentation tool will be interoperable with the radiation oncologists’ softwares to perform flap segmentation in a de-escalation phase III trial “optiflap” where irradiation is customized to target the junction between the flap and the native tissues rather than the whole flap. In routine practice, automatic flap delineation may be used with further validation by referring radiation oncologists, as it is done with automatic atlases.

## Data Availability

The datasets analyzed during the current study are not publicly available due to confidentiality agreements, institutional policies, and the sensitive nature of patient data, but are available from the corresponding author on reasonable request.
